# Editorial: Expanding therapeutic horizons with non-invasive vagus nerve stimulation

**DOI:** 10.3389/fnins.2026.1803982

**Published:** 2026-03-02

**Authors:** Claire-Marie Rangon, Jason Chen, Eric Azabou, Peter Staats

**Affiliations:** 1 SMART-VNS Platform (Structured Multidisciplinary platform to Advance Research on Therapy of Vagus Nerve Stimulation), Raymond Poincaré Hospital, AP-HP GHU Paris Saclay, Garches, France; 2 Clinical Neurophysiology and Neuromodulation Unit, Department of Physiology, Raymond Poincaré Hospital, AP-HP GHU University Paris Saclay, Garches, France; 3 Inserm UMR 1173 Infection and Inflammation (2I), University of Versailles Saint-Quentin en Yvelines (UVSQ), Paris-Saclay University, Garches, France; 4 Vagus Nerve Society, Atlantic Beach, FL, United States; 5 George Washington University School of Medicine, Washington, DC, United States; 6 National Spine and Pain Centers, Atlantic Beach, FL, United States

**Keywords:** cognitive functions, diabetes mellitus, gut–brain, insomnia, melatonin, neurodevelopmental disorders, non-invasive vagus nerve stimulation, surgery

The nervous system is fundamentally an electrical organ. Long before the molecular era, early physiologists recognized that bioelectric signals govern sensation, movement, and autonomic regulation. Today, advances in neuroscience, engineering, and clinical medicine are allowing us to return—more precisely and intentionally—to electricity as both a tool for understanding disease and a means to optimize human health and resilience. This Research Topic of *Frontiers in Neuroscience*, which we are honored to co-lead, explores the expanding therapeutic horizons of non-invasive vagus nerve stimulation (nVNS) as a cornerstone of this emerging bioelectric revolution.

The vagus nerve occupies a singular position at the intersection of brain, body, and environment. As the principal conduit of bidirectional communication between the central nervous system and peripheral organs, it integrates autonomic control, immune modulation, metabolic regulation, circadian biology, and emotional state. Non-invasive approaches to vagus nerve stimulation now allow us to access this critical pathway safely, repeatably, and at scale—opening new possibilities not only for treating disease, but for restoring physiologic balance and promoting wellness. The contributions in this Research Topic reflect the remarkable breadth of conditions influenced by vagal signaling.

First and foremost, the anti-inflammatory effect of vagus nerve stimulation, underscored more than 25 years ago by Kevin Tracey's team in Nature (Borovikova et al., 2000) is still making the front page of this prestigious journal (Jin et al., 2024). Initially invasive (VNS), devices have grown non-invasive (nVNS), becoming putatively available for a large number of patients unresponsive to conventional anti-inflammatory treatments. Far beyond the Cholinergic anti-inflammatory pathway (CAP) and neuro-immune reflexes, Liu et al. bring an up-to-date review of the mechanistic insights, including microbiota-vagus and mitochondria-vagus interactions, as well as future perspectives of nVNS, notably regarding precision neuromodulation (targeting distinct portions of the vagus nerve) and combination therapies, as seen below (Zhang, Ma et al.).

The anti-inflammatory properties of non-invasive vagus nerve stimulation are a recurring theme throughout the issue. Gargus et al. further examine neurodevelopmental disorders through the lens of early-life neuro-inflammation mediated by microglia, autonomic imbalance and disrupted neural connectivity. Non-invasive neuromodulation of brain plasticity offers a particularly compelling strategy in these populations, given its favorable safety profile and capacity for long-term use, not only regarding the neurological issues (epileptic encephalopathies) but also the psychiatric outcome (Autism Spectrum Disorders, Attention-Deficit/Hyperactivity Disorder or schizophrenia are tackled).

Three other articles (two pilot studies and one protocol) further explore the role of nVNS in cognitive function in adult and elderly patients. The first article is a promising single-blind, sham-controlled, crossover study assessing the effect of off-line electrical transcutaneous auricular vagus nerve stimulation (taVNS) on a short-term memory task, suggesting an improvement during the encoding phase of the task (Fisicaro et al.). The second one suggests that Vagus Nerve Magnetic modulation (VNMM) may be even more efficient than taVNS during dual-task paradigms, using functional near-infrared spectroscopy (fNIRS) (Wang et al.). The third one describes a protocol for a randomized controlled trial investigating the effectiveness of a 4 week-treatment of combined rTMS and taVNS on 88 participants with mild cognitive impairment further followed up during 4 weeks (Zhang, Ma et al.). These findings suggest applications that extend beyond traditional neurological disease into cognitive optimization and healthy aging.

Similarly, emerging data in sleep regulation (Zhou et al.) and melatonin secretion (Zhang, Zou et al.) point to vagal modulation as a means of restoring circadian harmony—an increasingly urgent need in a world characterized by sleep disruption and comorbid chronic illness involving gut–brain interaction like dyspepsia (Zhou et al.) or blood glucose regulation (Zhang, Ma et al.). By modulating vagal afferent and efferent pathways, nVNS demonstrates the potential to recalibrate gastric motility, visceral sensitivity, and central processing of interoceptive signals. These studies underscore the vagus nerve's role as a therapeutic bridge between neuroscience and gastroenterology.

Cardiometabolic health represents another critical frontier. Contributions addressing atrial fibrillation (Jiang et al.) and type 2 diabetes (Staats et al.) illustrate how vagal tone can influence cardiac electrophysiology, insulin sensitivity, and metabolic homeostasis. These findings challenge the traditional separation of neurological and systemic disease, reinforcing the concept that combined neuromodulation can serve as a unifying long-term therapeutic strategy across organ systems (Staats et al.), contrary to new drugs like Glucagon Like Peptide-1 agonists whose effects do not last (Wilding et al., 2022).

Last but not least, nVNS holds promise for not only chronic disorders but also for acute surgery like post-operative pain management of open fractures, a case report by (Li et al.) or post-operative fatigue syndrome in particularly frail elderly patients with cancer, a clinical hypothesis study by Yin et al., as a complementary strategy.

Collectively, these articles signal a paradigm shift. Non-invasive vagus nerve stimulation is no longer confined to a narrow set of indications; it is emerging as a platform technology for bioelectronic medicine. Importantly, this field invites a new model of care—one that complements pharmacology rather than replaces it, prioritizes physiologic regulation over symptom suppression, and emphasizes prevention, resilience, and systems-level health.

As we look to the future, several challenges and opportunities remain. Rigorous mechanistic studies, standardized stimulation parameters, and large-scale clinical trials will be essential. Equally important is the integration of nVNS into real-world settings, including home-based care and wellness applications, where its non-invasive nature offers unique advantages.

We hope this Research Topic will inspire neuroscientists, clinicians, engineers, and policymakers alike to reimagine what is possible when electricity is harnessed not only to treat disease, but to better understand the fundamental language of the nervous system, further blurring the boundaries between mental health, immune regulation, and systemic chronic illness. The bioelectric revolution is not on the horizon—it is already underway.
